# A spatial transcriptomics platform for characterizing cellular vulnerability and molecular changes in AD‐affected human brain tissue

**DOI:** 10.1002/alz.087488

**Published:** 2025-01-03

**Authors:** Jennie Close

**Affiliations:** ^1^ Allen Institute for Brain Science, Seattle, WA USA

## Abstract

**Background:**

Alzheimer’s disease is defined by deposition of pathological proteins in spatially reproducible patterns within the tissue of affected individuals. Prominent pathological protein species found in Alzheimer’s disease are amyloid beta plaques and phospho‐tau tangles, which spread through a succession of brain areas. As this pathology spreads and worsens, it is accompanied by glial reactivity and activation and neuronal loss. Characterization of cell types changing or lost during disease progression and where those changes occur will be important for finding mechanisms of disease onset and progression.

**Method:**

snRNAseq analysis from Alzheimer’s donors was used to determine which cell types are present and what changes in gene expression, if any, can be observed at the highest resolution of cell type identity: the supertype. We mapped these supertypes using highly multiplexed spatial transcriptomics on the same donor set to determine where these supertypes are in the tissue, where vulnerable types are lost at which stages, and whether gene expression changes can be observed in specific types near pathology.

**Result:**

We have found that certain vulnerable cellular supertypes are more likely to be affected early, and a reproducible sequence of neuronal loss follows with increasing pathology. Surprisingly, one of the first neuronal subtypes to be affected are somatostatin inhibitory neurons, followed by L2/3IT excitatory neurons and parvalbumin inhibitory neurons. We have corroborated these results in human donor tissue using spatial transcriptomics to map and characterize loss of abundance of vulnerable cell types. We find that vulnerable neuronal types are predominately localized in supragranular layers.

**Conclusion:**

Spatial methods are key to relating these neuronal vulnerabilities with pathological protein locations and specific locations within affected tissue. We have adapted spatial profiling and analysis methods to characterize the spatial relationships between specific neuronal types, amyloid plaques, tau tangles, and observed changes in gene expression. These data will offer novel insight into cell and molecular changes occurring at each phase of the disease and may inform the development of promising therapeutic interventions.

